# New Briarane Diterpenoids from the Gorgonian Coral *Junceella juncea*

**DOI:** 10.3390/md10061321

**Published:** 2012-06-07

**Authors:** Jiun-Yang Chang, Chia-Ching Liaw, Ahmed Eid Fazary, Tsong-Long Hwang, Ya-Ching Shen

**Affiliations:** 1 School of Pharmacy, College of Medicine, National Taiwan University, Jen-Ai Rd. Sec. 1, Taipei 101-200, Taiwan; Email: nomatter2200@hotmail.com (J.-Y.C.); biogodas@hotmail.com (C.-C.L.); aefazary@gmail.com (A.E.F.); 2 Institute of Marine Biotechnology and Resources, National Sun Yat-sen University, 70 Lien-Hai Road, Kaohsiung 80424, Taiwan; 3 Graduate Institute of Natural Products, Chang Gung University, Taoyuan 333, Taiwan; Email: htl@mail.cgu.edu.tw

**Keywords:** *Junceella juncea*, briaranes, anti-inflammatory activity

## Abstract

Chemical investigation of *Junceella juncea* has resulted in the isolation of three new briaranes designated juncenolides M–O (**1**–**3**). The structures of these compounds were determined by spectroscopic analysis including 2D-NMR (COSY, HMBC and NOESY) and HRMS. Compound **1 **is a new chlorinated briarane while compound **3** contains a rare methyl ester at C-16. The anti-inflammatory activities tested on superoxide anion generation and elastase release by human neutrophils in response to FMLP/CB were evaluated.

## 1. Introduction

**Figure 1:**
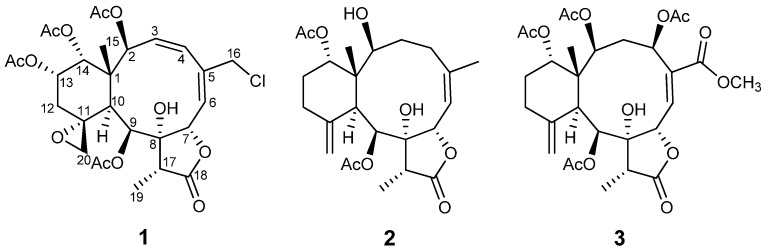
Structures of compounds **1**–**3**.

Gorgonian corals of the genus *Junceella* (Ellisellidae) are common in subtropical and tropical waters in a number of places around the world, such as the South China Sea and Indo-Pacific Ocean, and are well known as a source of highly oxidized diterpenes of the briarane class (3,8-cyclized cembranoids) [1]. Many *in vitro* and *in vivo* studies on diterpenes isolated from gorgonians showed a variety of biological activities including anti-tumor, anti-inflammatory, antiplasmodial, antibacterial, antiviral, antimalarial and antioxidant, as well as ecologically relevant activities such as fish-feeding deterrence. Diterpenes isolated from gorgonian corals have a large structural diversity with 40 different diterpene classes being represented [2]. Recently, three new 8-hydroxybriarane diterpenoids (junceols A–C) and four new briarane diterpenoids (juncenolides H–K) were reported from a chemical investigation of *Junceella juncea* Pallas collected off the southern Taiwan coast, and some of these metabolites were found to exhibit inhibitory effects on superoxide anion generation and elastase release by human neutrophils [3,4]. Fourteen new briarane diterpenes, juncins O–ZII, were isolated from the EtOH/CH_2_Cl_2_ extract of a South China Sea sample of *J. juncea* and some of them have been shown to exhibit potent antifouling and antifeedant activities [5,6,7]. A bioassay-guided fractionation of the acetone extract of a Taiwanese collection of *J. juncea* led to the identification of seven new diterpenoids, juncenolides A–G [8,9,10,11]. Moreover, a chemical investigation of the Indian Ocean gorgonian *J. juncea* resulted in the isolation of eight new briarane-type diterpenoids, juncins G–N [12,13,14]. A new briarane diterpenoid with antifungal activity was also isolated [15]. In continuation of our research programs oriented towards discovering new metabolites from the gorgonians collected off Taiwanese waters, we reinvestigated *J. juncea*. Examination of different chromatographic fractions of an AcOEt-soluble extract of the Taiwanese *J. juncea* Pallas resulted in the isolation of three new briaranes, designated juncenolides M–O (**1**–**3**) (Figure 1). Their structures were elucidated through detailed spectroscopic analyses, mainly 2D NMR experiments (^1^H, ^1^H COSY, HQMC, HMBC). The relative stereochemistry of the chiral centers and the geometry of the double bonds were deduced from NOESY spectra. 

## 2. Results and Discussion

**Figure 2:**
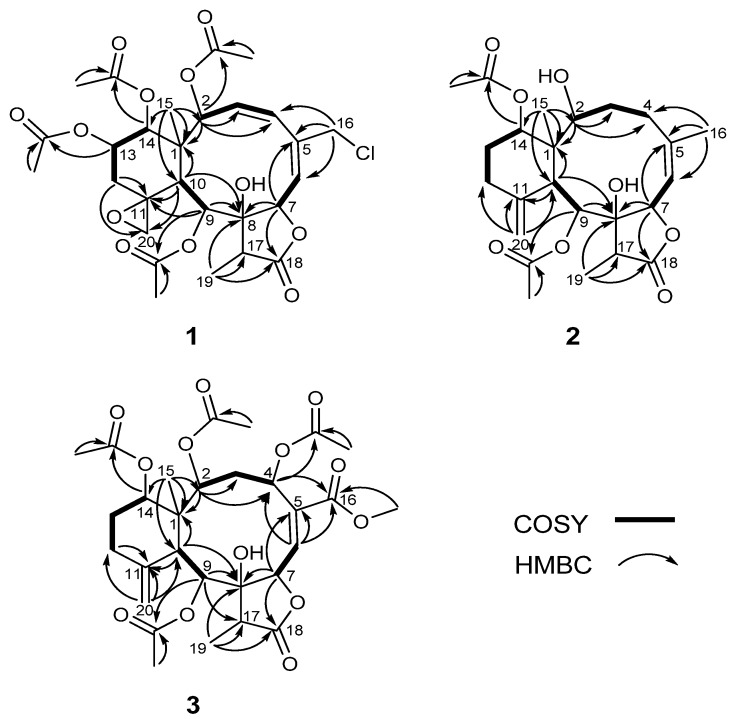
Key ^1^H–^1^H COSY and HMBC correlations of **1**–**3**.

Compound **1** was isolated as a colorless amorphous solid. The molecular formula was determined to be C_28_H_35_ClO_12_ (11 degrees of unsaturation) from the HR-ESI-MS data (*m/z* 621.1711 ([M + Na]^+^)), which also showed a M + 2 peak at *m/z* 623.1685 (3:1), indicating the presence of one chlorine atom. Its IR bands revealed the presence of a hydroxyl group (3402 cm^−1^), a five-membered lactone (1779 cm^−1^) and ester groups (1741 cm^−1^). ^1^H- and^ 13C-NMR data^ (Table 1 and Table 2) indicated the presence of four acetates (*δ*_H_ 2.18, s; 2.07, s; 1.97, s; 1.95, s) and *δ*_C_ 170.2 × 2; 170.0 × 2; 21.6; 21.3; 21.0; 20.8, one carbonyl carbon (*δ*_C_ 175.3), and two double bonds (*δ*_C_ 126.1; 128.1; 131.7; 140.0), suggesting four rings in the structure. The protons of CH_2_-20 (*δ*_H_ 3.54, br. s; 2.74, br. *s*), its corresponding carbon (*δ*_C_ 50.2) and the quaternary carbon at *δ*_C _58.1, were assigned to an exocyclic epoxide [16]. The presence of a γ-lactone ring was ascertained by the carbonyl carbon at *δ*_C_ 175.3 (C-18), and the *O*-bearing carbons at *δ*_C_ 78.5 (C-7) and 81.2 (C-8), and confirmed the HMBC correlations (Figure 2) of Me-19/C-8, C-17, C-18 and C-7/C-8, C-18 [16,17]. Four OAc groups were attached to C-2, C-9, C-13 and C-14 by the observation of HMBC correlations (Figure 2). A tertiary methyl signal (*δ*_H_ 1.09, Me-15) correlated with a quaternary carbon (C-1), two oxymethines at *δ*_C_ 74.2 and 73.8, and the CH at *δ*_C_ 41.4, (C-10), implying oxygenation at C-2 and C-14. HMBC correlations of Me-15/C-1, C-2, C-10, C-14, CH_2_-16/C-4, C-5, C-6, CH-7/C-5, C-8, C-18, CH-9/C-8, C-11, and CH-10/C-1, C-8, C-11, C-20, as well as ^1^H–^1^H COSY connectivities between CH-2/CH-3/CH-4, CH-6/CH-7, CH-9/CH-10, CH_2_-12/CH-13/CH-14 (Figure 1), suggested that compound **1** possesses 8-hydroxybriarane-type diterpenoid skeleton together with an exocyclic epoxy group which was corroborated by HMBC correlations of CH_2_-12/C-11, C-20. 

**Table 1:** ^1^H NMR Data of Compounds **1**–**3**. *δ* in ppm, *J* in Hz.

Position	1 ^a^	2 ^b^	3 ^b^
2	5.37 (d, *J* = 9.6)	4.94 (overlap)	4.86 (d, *J* = 7.6)
3	5.62 (t, *J* = 9.6)	1.74–1.78 (m)	2.13–2.16 (m)
		2.46–2.50 (m)	2.72 (t, *J* = 10.0)
4	6.35 (d, *J* = 9.6)	2.19–2.15 (m)	5.91–5.94 (m)
		2.64 (br. d, *J* = 13.6)	
6	6.00 (d, *J* = 9.0)	5.67 (d, *J* = 10.4)	7.06 (d, *J* = 10.0)
7	4.95 (d, *J* = 9.0)	5.26 (d, *J* = 10.4)	5.62 (d, *J* = 10.0)
9	4.71 (d, *J* = 4.8)	5.31 (d, *J* = 6.0)	5.56 (d, *J* = 2.8)
10	3.04 (d,*J* = 4.8)	3.45 (d, *J* = 6.0)	3.25 (d, *J* = 2.8)
12	1.34–1.38 (m)	2.16–2.19 (m)	2.18–2.23 (2H, m)
	2.48 (d, *J* = 14.0)	2.34–2.36 (m)	
13	4.96 (overlap)	1.82–1.86 (m)	1.80–1.87 (2H, m)
		1.97–1.99 (m)	
14	5.20 (br. s)	4.59 (br. s)	4.69 (br. s)
15	1.09 (s)	1.12 (s)	1.03 (s)
16	4.58 (2H, s)	2.05 (s)	-
17	2.26 (q, *J* = 6.9)	2.46 (q, *J* = 6.8)	2.63 (q, *J* = 6.8)
19	1.13 (d, *J* = 6.9)	1.11 (d, *J* = 6.8)	1.19 (d, *J* = 6.8)
20	2.74 (br. s)	4.92 (s)	4.95 (s)
	3.54 (br. s)	5.05 (s)	5.05 (s)
AcO-2	1.95 (s)	-	1.97 (s)
AcO-4	-	-	2.06 (s)
AcO-9	2.18 (s)	2.20 (s)	2.21 (s)
AcO-13	2.07 (s)	-	-
AcO-14	1.97 (s)	1.92 (s)	1.90 (s)
OMe-16	-	-	3.83 (s)

^a^ Recorded in CDCl_3_ at 300 MHz; ^b^ Recorded in CDCl_3_ at 400 MHz.

**Table 2:** ^13^C-NMR Data of Compounds **1**–**3**.*δ* in ppm.

Position	1 ^a^	2 ^b^	3 ^b^
1	46.5 (s)	46.9 (s)	47.8 (s)
2	74.2 (d)	75.7 (d)	72.1 (d)
3	131.7 (d)	31.3 (t)	37.3 (t)
4	128.1 (d)	29.2 (t)	67.4 (d)
5	140.0 (s)	144.8 (s)	136.8 (s)
6	126.1 (d)	120.5 (d)	139.1 (d)
7	78.5 (d)	77.8 (d)	76.7 (d)
8	80.8 (s)	83.0 (s)	82.9 (s)
9	64.3 (d)	71.3 (d)	72.7 (d)
10	35.7 (d)	41.8 (d)	42.6 (d)
11	58.1 (s)	150.8 (s)	150.5 (s)
12	34.3 (t)	26.3 (t)	29.2 (t)
13	67.7 (d)	26.9 (t)	27.5 (t)
14	73.8 (d)	74.5 (d)	74.0 (d)
15	14.4 (q)	15.4 (q)	14.4 (q)
16	44.7 (t)	27.2 (q)	166.6 (s)
17	43.9 (d)	42.5 (d)	43.3 (d)
18	175.3 (s)	175.9 (s)	175.3 (s)
19	6.3 (q)	6.5 (q)	6.4 (q)
20	50.2 (t)	113.3 (t)	113.3 (t)
AcO-2	170.2 (s)	-	169.9 (s)
	20.8 (q)		20.8 (q)
AcO-4	-	-	169.6 (s)
			21.2 (q)
AcO-9	170.0 (s)	169.3 (s)	169.2 (s)
	21.6 (q)	21.7 (q)	21.7 (q)
AcO-13	170.0 (s)	-	-
	21.3 (q)		
AcO-14	170.2 (s)	170.4 (s)	170.5 (s)
	21.0 (q)	21.2 (q)	21.1 (q)
OMe-16	-	-	52.8 (q)

^a^ Recorded in CDCl_3_ at 75 MHz; ^b^ Recorded in CDCl_3_ at 100 MHz.

**Figure 3:**
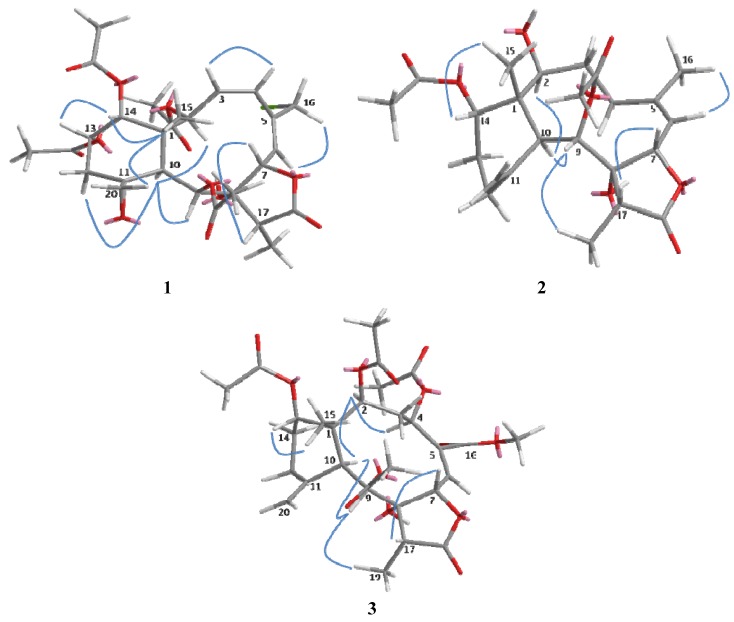
Key NOESY correlations of compounds **1**–**3** in molecular modeling.

The relative configuration of **1** was determined on the basis of NOESY experiment, MM2 minimized energy calculated molecular modeling (Figure 3), and comparison with other naturally occurring briarane diterpenoids. Briarane-type diterpenoids were previously reported to contain the Me-15 in the β-orientation and H-10 in the α-orientation. As expected, there is no NOE correlation between CH-10 and Me-15. The orientations of CH-10 and the methyl group Me-15 should be opposite. According to MM2 study, we thus concluded that compound **1 **had the Me-15 in the β-orientation and CH-10 in the α-orientation as reported [18]. NOESY correlations of CH-10/H-2, CH-9, CH_α_-12, Me-15/CH_2_-20, CH-14, CH_2_-20/CH-13, CH_β_-12 and CH-14/CH-13 suggested the β-orientation of CH-13 and CH-14 and α-orientation of CH-2 and CH-9, a β-oriented exocyclic epoxy group attached to cyclohexane moiety as previously assigned from the ^1^H- and ^13^C NMR data of C-11 and C-20 [19] and the α-orientation of CH-2. The *cis* configuration of the C-3/C-4 double bond was suggested by the NOESY correlations (Figure 3) between CH-3/CH-4 and the *J* value (9.6 Hz). Compound **1** appears to be the chlorinated derivative of juncenolide B isolated from the same species previously [9]. They have the same configuration of chiral centers. Based on the above interpretation, compound **1** is a new chlorinated briarane-type diterpene designated juncenolide M. 

Compound **2**, isolated as a colorless solid, and had a molecular formula of C_24_H_34_O_8_, deduced from HR-ESI-MS at *m/z* 473.2155 ([M + Na]^+^), showing eight degrees of unsaturation. The presence of hydroxyl, a γ-lactone ring, and ester groups were consistent with IR absorptions at 3481, 1772 and 1732 cm^−1^. The ^1^H and^ 13C NMR^ (Table 1 and Table 2), revealed the presence of an exomethylene (*δ*_C_ 150.8, 113.3), one trisubstituted double bond (*δ*_C_ 144.8, 120.5), two OAc carbonyl (C=O) (*δ*_C_ 170.4, 169.3) and one γ-lactone carbonyl (C=O) (*δ*_C_ 175.9), which accounted for five degrees of unsaturation and were suggestive of a tricyclic briarane bearing a γ-lactone ring. The carbonyl signal at *δ*_C_ 175.9 (C-18) was ascribed to a γ-lactone ring with the oxymethine at *δ*_C_ 78.3 (C-7) and the *O*-bearing quaternary carbon at *δ*_C_ 82.2 (C-7). The proton singlets at *δ*_H_ 5.05 and 4.92 (*δ*_C_ 113.3) were assigned to the exocyclic methylene group and correlated to C-10, C-12, and C-11 in the HMBC spectrum (Figure 2), suggesting the presence of a C-11/C-20 double bond. HMBC correlations of Me-15/C-1, C-2, C-14, C-10, Me-16/C-4, C-5, C-6, CH-7/C-5, C-8, C-18, CH-9/C-8, CH-10/C-11, C-1, C8 and the ^1^H, ^1^H COSY correlations of CH-2/CH_2_-3/CH_2_-4, CH-6/CH-7, CH-9/CH-10, CH_2_-12/CH_2_-13/CH-14 revealed the tricyclic skeleton of **2**. Furthermore, two OAc groups positioned at C-9 and C-14 were established by the key correlations observed in the HMBC spectrum of **2. **NOESY experiment revealed that the absence of correlation between and suggested orientation of and disposition of compound **2**. The NOESY correlations (Figure 3) between Me-15/CH-14; CH-10/CH-2, CH-9, CH-9/Me-19, and CH-7/CH-17 were in agreement with the β-orientation of CH-7, CH-14 and CH-17, and α-orientation of CH-2, CH-9 and Me-19. Therefore, compound **2 **is a new tricyclic briarane bearing a γ-lactone ring, and was given the name of juncenolide N.

The molecular formula of **3 **was established as C_29_H_38_O_13_ from the molecular peak at *m/z* 617.2212 [M + Na]^+^ in the HR-ESI-MS. The NMR data (Table 1 and Table 2) revealed the basic features of a 8-hyoxybriarane type diterpenoid with a γ-lactone, one exomethylene double bond, one trisubstituted double bond (Table 1 and Table 2), and four acetate esters. The C-20/C-11 exomethylene double bond was assigned with the aid of HMBC correlations of CH_2_-20/C-10, C-11, C-12. The signal of CH-6 showed correlations to C-4, C-5 and C-16 in HMBC, revealing the C-5/C-6 trisubstituted double bond, and a COOMe group attached to C-5 (Figure 2). The four acetates were deduced to be located at C-2, C-4, C-9, and C-14 by HMBC correlations of the oxymethines at *δ*_H_ 4.86 (CH-2), 5.93 (CH-4), 5.56 (CH-13), and 4.69 (CH-14) to their respective acetate carbonyls. The ^1^H,^1^H COSY connectivities (Figure 2) of CH-2/CH_2_-3/CH-4, CH-6/CH-7, CH-9/CH-10, CH_2_-12/CH_2_-13/CH-14, as well as HMBC correlations of Me-15/C-1, C-2, C-10, C-14, CH-4/C-16, CH-7/C-8, C-18, CH-9/C-8, C-17, CH-10/C-1, C-8, C-11, CH_2_-12/C-11 and Me-19/C-8, C-17, C-18, confirmed the tricyclic skeleton of **3**. The NOESY experiments (Figure 3) showed the relative configuration of compound **3**. Due to the α-orientation of CH-10, the methyl group Me-15 at the ring junction should be β-oriented as no NOE correlation was observed between CH-10 and Me-15. NOESY spectrum clearly displayed the interactions between Me-15/CH-14, CH-10/CH-9, CH-2, CH-2/CH-4, and CH-9/CH-19, indicating that the OAc at C-2, C-4 and C-9 are β-oriented, whereas the OAc at C-14 is in the α-position. Thus, compound **3 **is a new briarane ester with a γ-lactone skeleton, and designated juncenolide O.

The isolated briaranes **1**–**3** were tested on inhibitory effects of superoxide anion generation and elastase release by human neutrophils in response to FMLP/CB at a concentration of 10 μg/mL. As illustrated in Table 3, compounds **2** and **3** showed moderate inhibitory activities against elastase release with 29.0 ± 5.6%, and 35.9 ± 7.4%, respectively. Furthermore, compound **3** also exhibited moderate inhibitory activity against superoxide anion with 27.6 ± 7.0%.

**Table 3:** Effects of compounds **1**–**3** on superoxide anion generation and elastase release by human neutrophils in response to FMLP/CB ^a^.

**Compounds**	**Superoxide anion**	**Elastase release**
	Inhibition (%) ^b^	Inhibition (%)
**1**	7.6 ± 2.8	15.9 ± 5.5
**2**	6.7 ± 2.9	29.0 ± 5.6
**3**	27.6 ± 7.0	35.9 ± 7.4
Genistein	65.0 ± 5.7	51.6 ± 5.9

^a^ Results are presented as mean ± S.E.M. (*n* = 3); ^b^ Percent of inhibition at 10 μg/mL.

## 3. Experimental Section

### 3.1. General

Column chromatography (CC); silica gel 60 (Merck, Darmstadt, Germany) and Sephadex LH-20 (Amersham Pharmacia Biotech AB, Uppsala, Sweden). Prep. TLC: pre-coated silica gel plates (Merck; silica gel 60 F-254, 1 mm). LiChrospher Si 60 (5 μm, 250-10, Merck) and LiChrospher 100 RP-18e (5 μm, 250-10, Merck) were used for NP-HPLC and RP-HPLC (Merck, Darmstadt, Germany), respectively. Spray reagent: *p*-anisaldehyde reagent with 5% H_2_SO_4_. Optical rotations: Jasco DIP-1000 polarimeter. UV Spectra: Hitachi U-3210 spectrometer; λ_max_ (log ε) in nm. IR Spectra: Hitachi T-2001 spectrometer; in cm^−1^. ^1^H-, ^13^C-NMR, COSY, HMQC, HMBC, and NOESY experiments: Bruker Avance 300 NMR spectrometer or Varian MR 400 NMR spectrometer, SiMe_4_ as internal standard; *δ* in ppm, coupling constants *J* in Hz. LRESIMS and HRESIMS: JEOL JMS-HX 110 mass spectrometer; in *m/z*.

### 3.2. Animal Material

The gorgonian *Junceella juncea* Pallas (Ellisellidae) was collected in Tai-Tong County, Taiwan, by scuba diving at a depth of 15 m, in November 2006. The fresh gorgonian was immediately frozen after collection and kept at −20 °C until processed. This species was identified by one of the authors (C.-C.L). A voucher specimen (WSG-5) was deposited in the School of Pharmacy, College of Medicine, National Taiwan University, Taiwan.

### 3.3. Extraction and Isolation

The outer grey layer of the gorgonian (1.4 kg, wet weight) was extracted with acetone (3 × 500 mL) at r.t., and the acetone extract was concentrated under vacuum. The crude extract (8 g) was partitioned between AcOEt and H_2_O (1:1). The AcOEt-soluble portion (4.9 g) was subjected to column chromatography (SiO_2_, *n*-Hexane/AcOEt 10:1–0:1; TLC (GF_254_) monitoring) giving fractions 1-16. Fr. 12 (195 mg) was subjected to a NP-HPLC (CH_2_Cl_2_/MeOH, 150:1), affording Fr. 12a (12 mg) which was further purified by RP-HPLC (MeOH/H_2_O/CH_3_CN, 70:25:5) yielding compound **1 **(6 mg). Fr. 16 (105 mg) was separated by NP-HPLC (CH_2_Cl_2_/MeOH, 80:1), giving Fr. 16a (38 mg) which was subjected to RP-HPLC (MeOH/H_2_O/CH_3_CN, 55:40:5), yielding Fr. 16b (10 mg) that was further purified by RP-HPLC (MeOH/H_2_O/CH_3_CN, 55:40:5) furnishing compounds **2 **(4 mg) and **3** (2 mg). 

Juncenolide M (**1**): colorless amorphous solid; [α]_D_^25^ = −42 (*c* 0.05, CH_2_Cl_2_); UV (MeOH): 221 (3.20); IR (neat): 3402 (OH), 2930, 2853, 1779 (C=O γ-lactone), 1741 (C=O ester) cm^−1^; ^1^H-NMR (300 MHz, CDCl_3_) data, see Table 1; ^13^C-NMR (75 MHz, CDCl_3_) data, see Table 2; HR-ESI-MS [M + Na]^+^ *m/z* 621.1711 (calcd. 621.1715, C_28_H_35_ClO_12_Na).

Juncenolide N (**2**): colorless amorphous solid; [α]_D_^25^ = −60 (*c* 0.05, CH_2_Cl_2_); UV (MeOH): 204 (3.90); IR (neat): 3481 (OH), 1772 (C=O γ-lactone), 1732 (C=O ester) cm^−1^; ^1^H-NMR (400 MHz, CDCl_3_) data, see Table 1; ^13^C-NMR (100 MHz, CDCl_3_) data, see Table 2; HR-ESI-MS [M + Na]^+^ *m/z* 473.2155 (calcd. 473.2151, C_24_H_34_O_8_Na).

Juncenolide O (**3**): colorless amorphous solid; [α]_D_^25^ = +4 (*c* 0.05, CH_2_Cl_2_); UV (MeOH): 220 (3.80), 205 (3.90); IR (neat): 3423 (OH), 3020, 2921, 2850, 1780 (C=O γ-lactone), 1738 (C=O ester) cm^−1^; ^1^H-NMR (400 MHz, CDCl_3_) data, see Table 1; ^13^C-NMR (100 MHz, CDCl_3_) data, see Table 2; HR-ESI-MS [M + Na]^+^ *m/z* 617.2212 (calcd. 617.2210, C_29_H_38_O_13_Na).

### 3.4. Anti-Inflammatory Assays

Neutrophils were obtained by means of dextran sedimentation and Ficoll centrifugation. Superoxide generation and elastase release were carried out according to a procedure described previously [20]. Superoxide anion production was assayed by monitoring the superoxide dismutase-inhibitable reduction of ferricytochrome *c*. Elastase release experiments were performed using MeO-Suc-Ala-Ala-Pro-Valp-nitroanilide as the elastase substrate. Genistein was used as a positive control.

## 4. Conclusions

Three new diterpenoids, named juncenolides M–O (**1**–**3**), were isolated from the Taiwanese gorgonian *Junceella juncea* Pallas. Compound **1** is a new chlorinated briarane, compound **2** is a new brierane with a free hydroxy at C-2, while compound **3** contains a rare methyl ester at C-5. The anti-inflammatory activities tested on superoxide anion generation and elastase release by human neutrophils in response to FMLP/CB were evaluated. As a result, compounds **2** and **3** showed moderate inhibitory activities against elastase release at 10 μg/mL.
